# Correction: Alcalá, S., et al. Targeting *SRC* Kinase Signaling in Pancreatic Cancer Stem Cells. *Int. J. Mol. Sci.* 2020, *21*, 7437

**DOI:** 10.3390/ijms21239215

**Published:** 2020-12-03

**Authors:** Sonia Alcalá, Víctor Mayoral-Varo, Laura Ruiz-Cañas, Juan Carlos López-Gil, Christopher Heeschen, Jorge Martín-Pérez, Bruno Sainz

**Affiliations:** 1 Department of Cancer Biology, Instituto de Investigaciones Biomédicas “Alberto Sols” (IIBM), CSIC-UAM, 28029 Madrid, Spain; sonia.alcala@uam.es (S.A.); vmayoral@iib.uam.es (V.M.-V.); lauraruiz@iib.uam.es (L.R.-C.); jclopez@iib.uam.es (J.C.L.-G.); jmartin@iib.uam.es (J.M.-P.); 2 Department of Biochemistry, Universidad Autónoma de Madrid (UAM), 28029 Madrid, Spain; 3 Chronic Diseases and Cancer, Area 3-Instituto Ramón y Cajal de Investigación Sanitaria (IRYCIS), 28034 Madrid, Spain; 4 Stem Cells & Cancer Group, Molecular Pathology Programme, Spanish National Cancer Research Centre (CNIO), 28029 Madrid, Spain; christopher.heeschen@icloud.com; 5 Center for Single-Cell Omics and Key Laboratory of Oncogenes and Related Genes, School of Medicine, Shanghai Jiao Tong University, Shanghai 200240, China

The authors recently reported on the potential of targeting SRC kinase signaling in pancreatic cancer stem cells [1]. During the preparation of this article, we submitted an incorrect version of Figure 3 [1] in which two β-actin housekeeping bands in Panel B were erroneously not included. Thus, Figure 3 [1] should be replaced with the following figure (Figure 1), which includes the excluded two β-actin housekeeping bands, images of the exact β-actin housekeeping bands used for the densitometric analyses reported in the original version, and an extended figure legend detailing which immunoblotted proteins share the same β-actin control bands, due to the fact that they originated from the same gel/membrane. We also provided a new Supplementary Material file where Figure S4 has been re-organized such that the uncropped and unprocessed images of the Western Blot images are now organized by gel so that shared β-actin housekeeping bands can be easily identified.

This error does not affect any of the data or conclusions in the article, and all densitometric analyses remain the same. The authors apologize for any confusion this error may have caused to the readers.

## Figures and Tables

**Figure 1:**
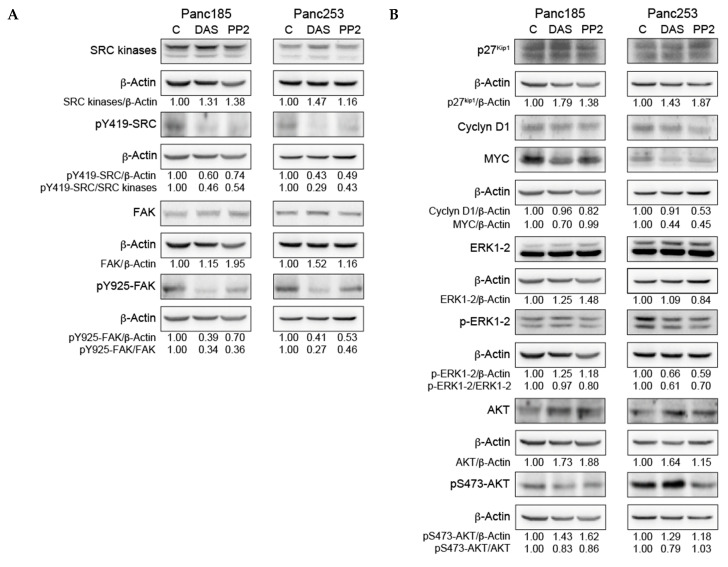
The effect of inhibition of *SRC* kinases on key signaling factors. (**A**) WB analysis of *SRC* kinases and pY419–*SRC* protein expression (top) or FAK and pY925–FAK (bottom) in control-, dasatinib (DAS)- or PP2-treated Panc185 or Panc253 PaCSCs. The indicated ratios for pY419–*SRC/SRC* kinases and pY925–FAK/FAK were determined, and the fold-changes are shown, setting diluent (C)-treated cells as 1.0. (**B**) WB analysis of p27^Kip1^, cyclin D1, MYC, ERK1-2, pERK1-2, AKT, or pS473–AKT protein levels in control-, DAS-, or PP2-treated Panc185 or Panc253 PaCSCs. The indicated ratios for p27^Kip1^/β-actin, cyclin D1/β-actin, MYC/β-actin, pERK1-2/ERK1-2, and pS473–AKT/AKT were determined and the fold-changes are shown, setting diluent (C)-treated cells as 1.0. Densitometric analysis of the indicated bands was determined using ImageJ software, and all values were normalized to β-actin values, which were included as a loading and normalization control. Four gels were run and transferred. Gel/membrane 1 was blotted for pY419–*SRC*, pY925–FAK, total ERK1-2, MYC and Cyclin D1 and thus share the same β-actin control. Gel/membrane 2 was blotted for SRC kinases, FAK and pERK1-2 and thus share the same β-actin control. Gel/membrane 3 was blotted for total AKT and β-actin. Gel/membrane 4 was blotted for pS473–AKT and p27 and thus share the same β-actin control. Uncropped and unprocessed images of Gels 1–4 can be found in Figure S4.
