# Supplementary material for: Targeting SRC Kinase Signaling in Pancreatic Cancer Stem Cells
Source: Int J Mol Sci. 2020 Oct 9;21(20):7437. doi: 10.3390/ijms21207437 (PMC7588004; doi:10.3390/ijms21207437)

Table S1 Detailed information for antibodies used

Name	Type	Supplier	Catalog #	Origin	Dilution Ratio
β-Actin	mouse monoclonal	Sigma-Aldrich (Merck)	A5441	San Luis, MI, USA	1:1000
$p27^{Kip1}$ (Clone G173-524)	mouse monoclonal	BD-Biosciences	#554069	Franklin Lakes, NJ, USA	1:1000
Cyclin D1 (H-295)	rabbit polyclonal	Santa Cruz Biotechnology, Inc.	sc-753	Dallas, TX, USA	1:500
Src-kinases (Src2)	rabbit polyclonal	Santa Cruz Biotechnology, Inc.	sc-18	Dallas, TX, USA	1:1000
pY418-SRC	rabbit polyclonal	Invitrogen	#44660	Camarillo, CA, USA	1:1000
FAK	rabbit polyclonal	Santa Cruz Biotechnology, Inc.	sc-557	Dallas, TX, USA	1:1000
Y925-FAK	rabbit polyclonal	Cell Signaling Technology	CTS#9101	Danvers, MA, USA	1:1000
ERK1/2	rabbit polyclonal	Santa Cruz Biotechnology, Inc.	sc-154	Dallas, TX, USA	1:1000
pT202/pY204-ERK1-2	rabbit polyclonal	Cell Signaling Technology	CST #9101	Danvers, MA, USA	1:1000
AKT	rabbit polyclonal	Santa Cruz Biotechnology, Inc.	sc-8312	Dallas, TX, USA	1:1000
pS473-AKT	rabbit polyclonal	Cell Signaling Technology	CST #9271	Danvers, MA, USA	1:1000
MYC	rabbit polyclonal	Santa Cruz Biotechnology, Inc.	sc-764	Dallas, TX, USA	1:1000
CD133-APC	mouse monoclonal	Miltenyi	130-111-080	Bergisch Gladbach, Germany	1:20
CXCR4-PE	mouse monoclonal	Miltenyi	130-117-354	Bergisch Gladbach, Germany	1:20

Figure S1 – *SRC* overexpression correlates with CSC-related pathways

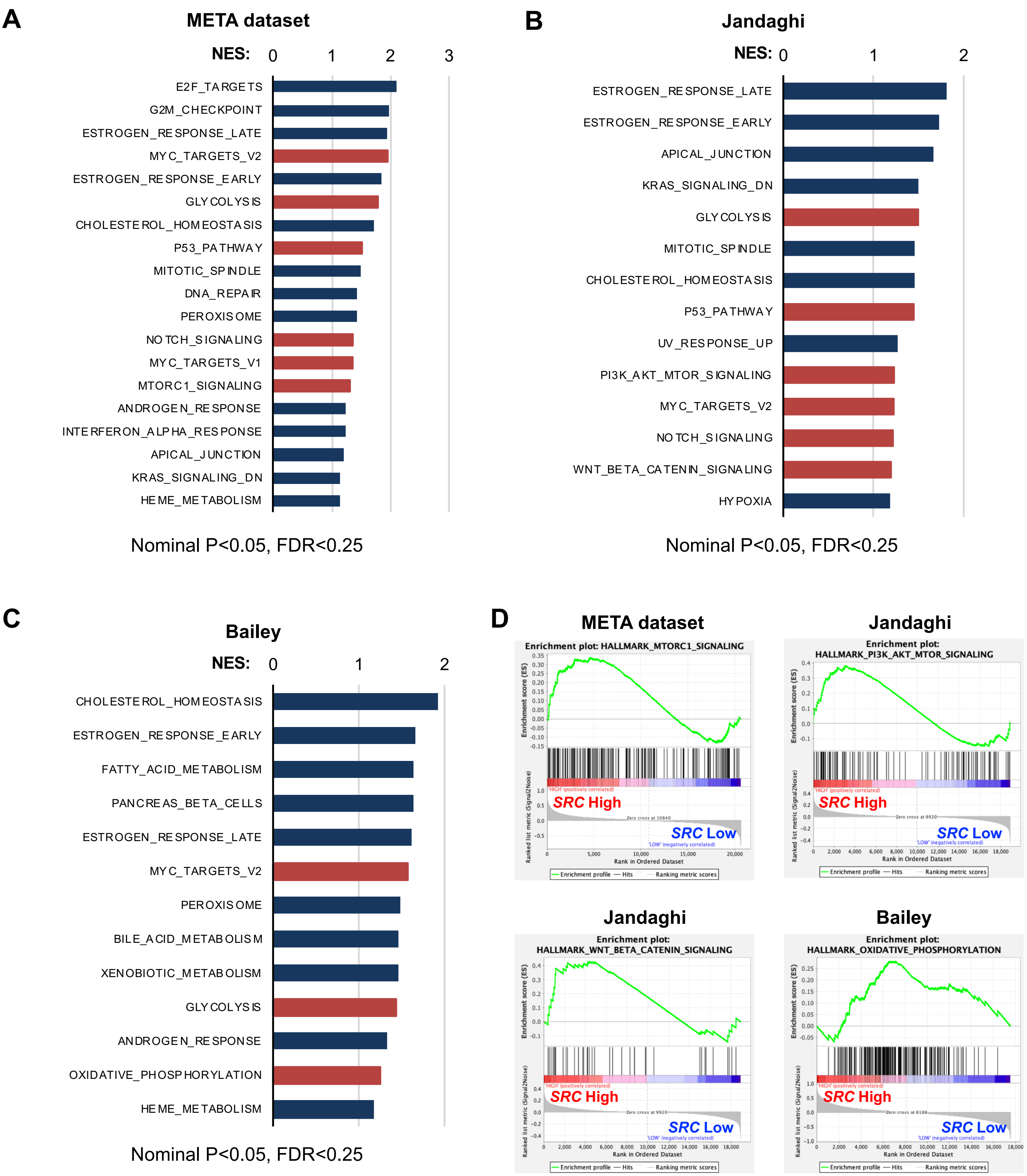

Figure S1. *SRC* overexpression correlates with CSC-related pathways. (**A-C**) Pathways enriched in the transcriptional profiles of tumors belonging to the top *SRC* high expression group, compared with the bottom expression group in the META (**A**), Jandaghi (**B**) or Bailey (**C**) datasets. A nominal p value of <0.05, FDR<25% is considered statistically significant. Shown are the NES values for each pathway using the Hallmark genesets. Stem-related pathways are shown in red. (**D**) Example enrichment plots for MTORC1, PI3K/AKT/MTOR, WNT/ß-catenin and Oxidative Phosphorylation signaling pathways from the indicated datasets.

Figure S2 – SRC kinase inhibitor-associated toxicity

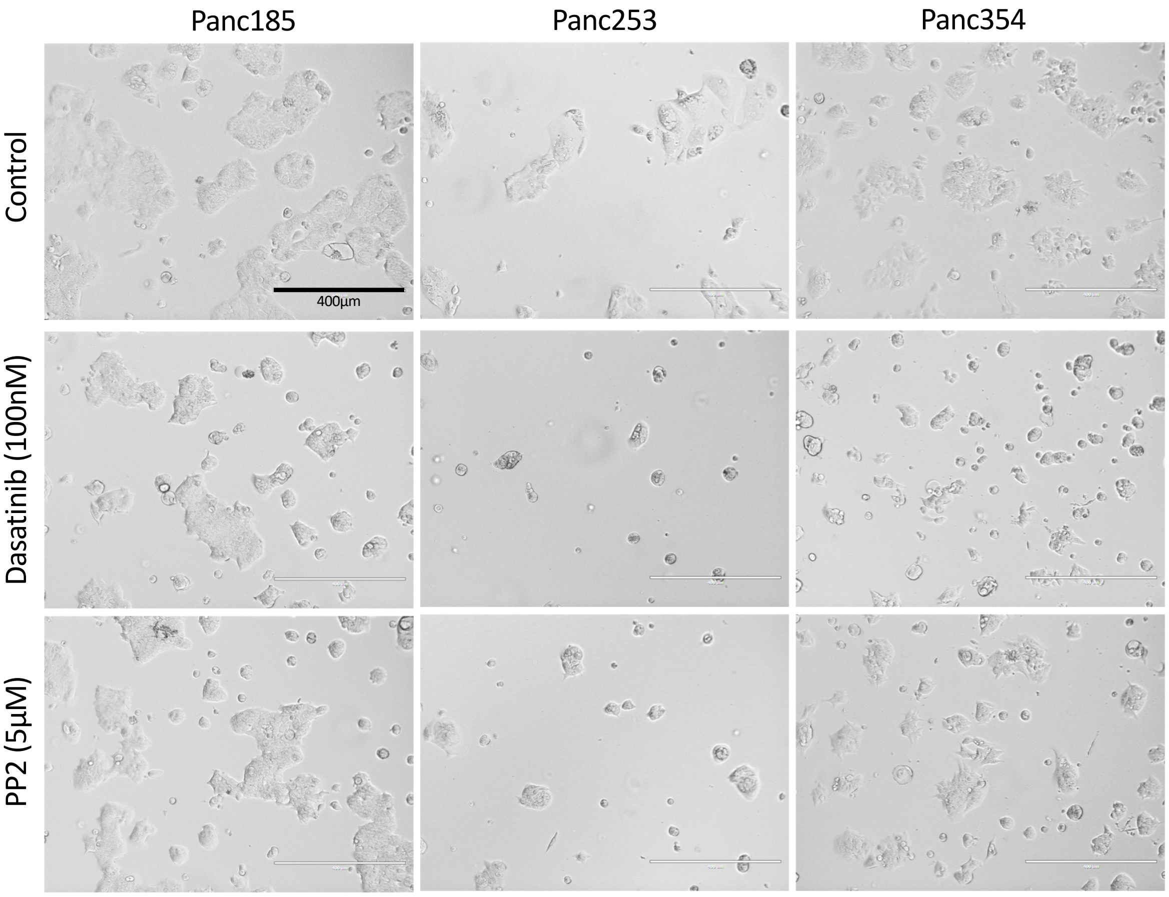

Figure S2. SRC kinase inhibitor-associated toxicity. Light micrographs of Panc185, Panc253 and Panc354 cell viability and growth following long term (7 day) treatment with Dasantinib or PP2 at the indicate concentrations.

Figure S3 – The effect of SRC kinase inhibition on FAK

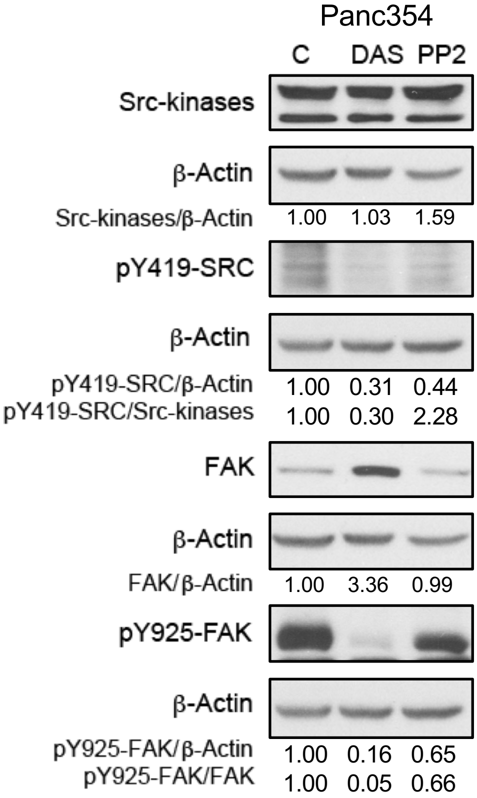

Figure S3. The effect of SRC kinase inhibition on FAK. WB analysis of SRC kinases and pY419-SRC protein expression (top) or FAK and pY925-FAK (bottom) in control-, PP2- or Dasatinib (DAS)-treated Panc185 or Panc253 PaCSCs. The indicated ratios for pY419-SRC/SRC-kinases and pY925-FAK/FAK were determined and shown are the fold-changes, setting control diluent(C)-treated cells as 1.0.

Figure S4 – Uncropped immunoblot images

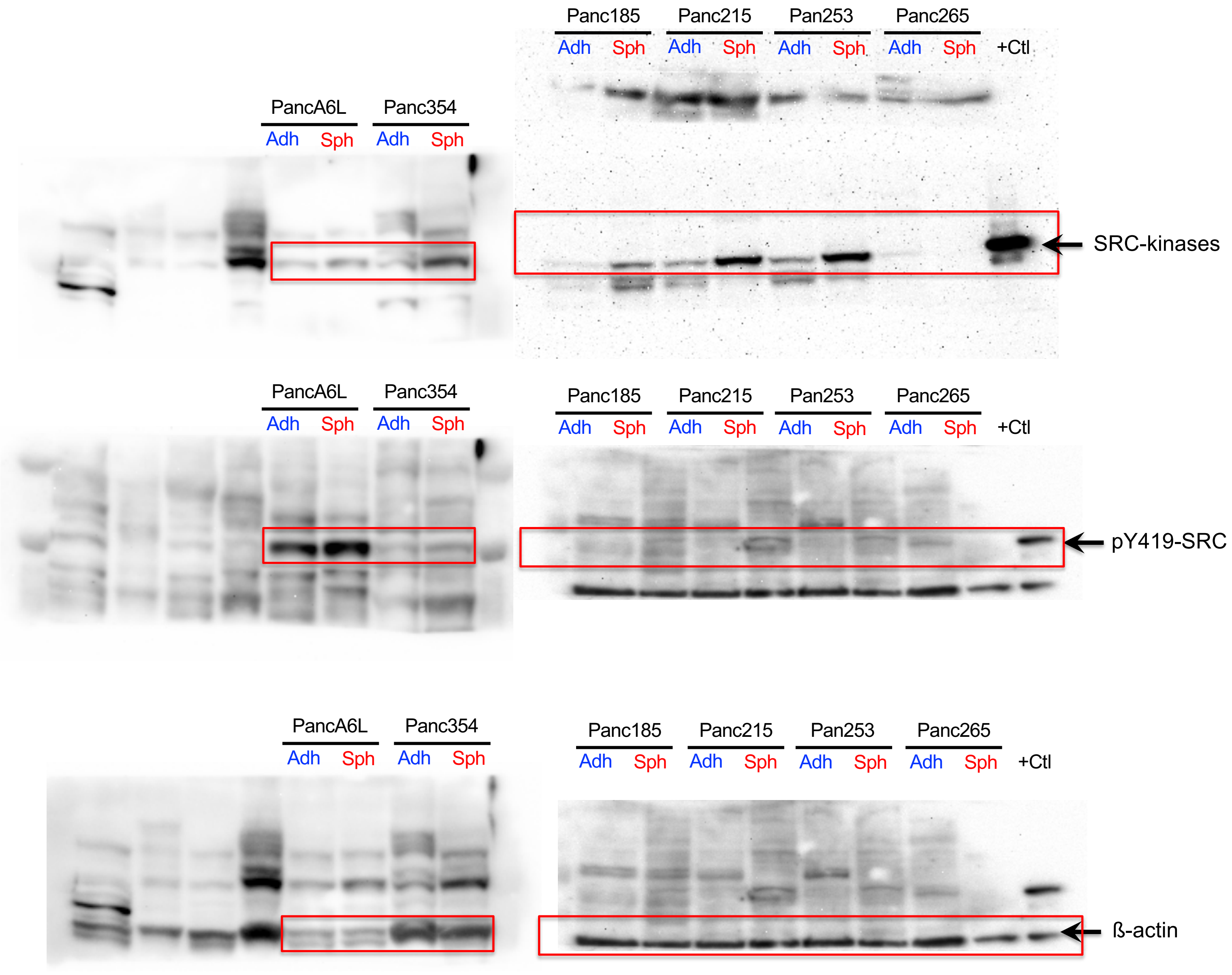

Figure S4 – Uncropped immunoblot images - continued

Unprocessed images for Figure 5

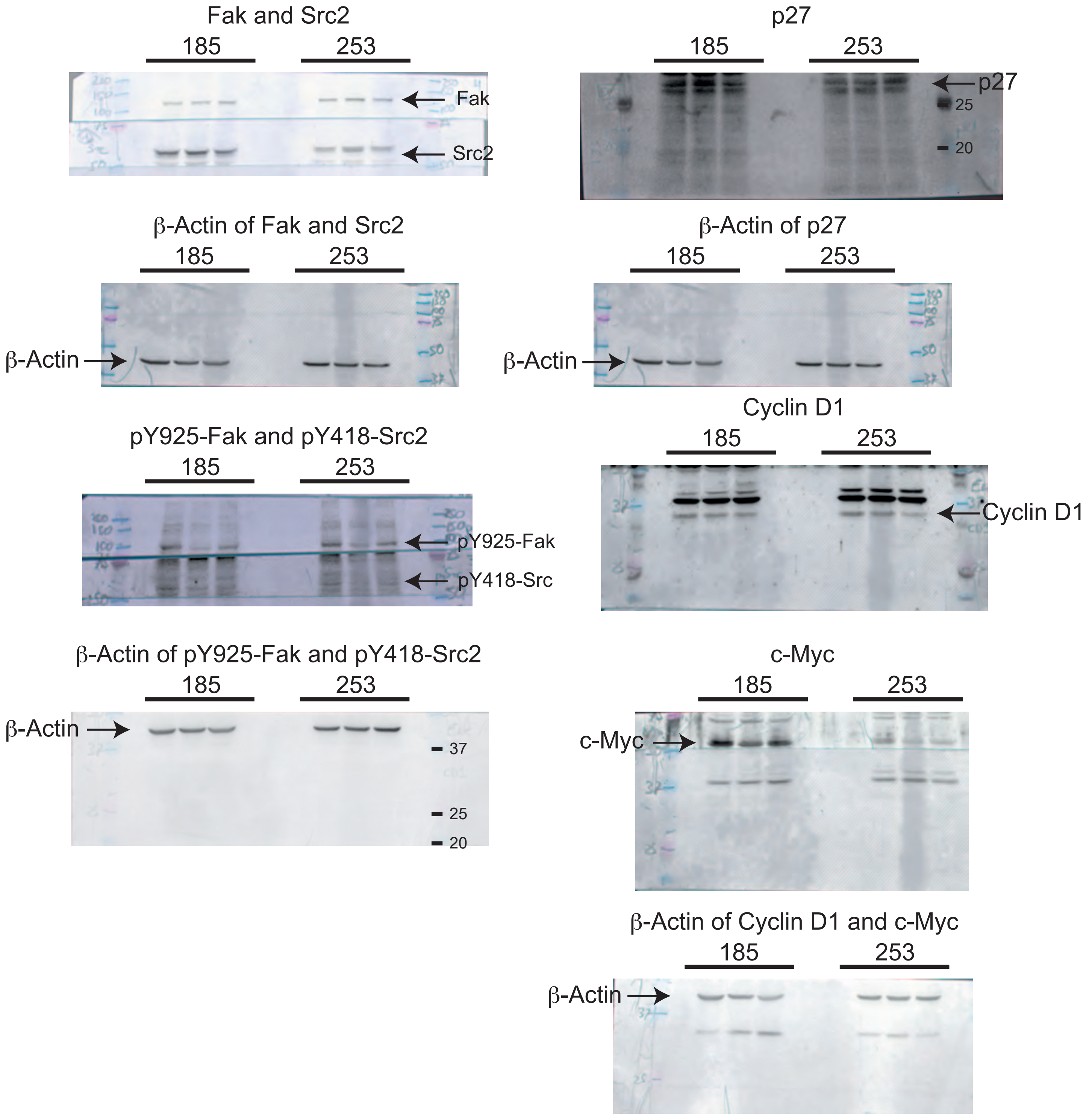

Figure S4 – Uncropped immunoblot images - continued

Unprocessed images for Figure 5 - continued

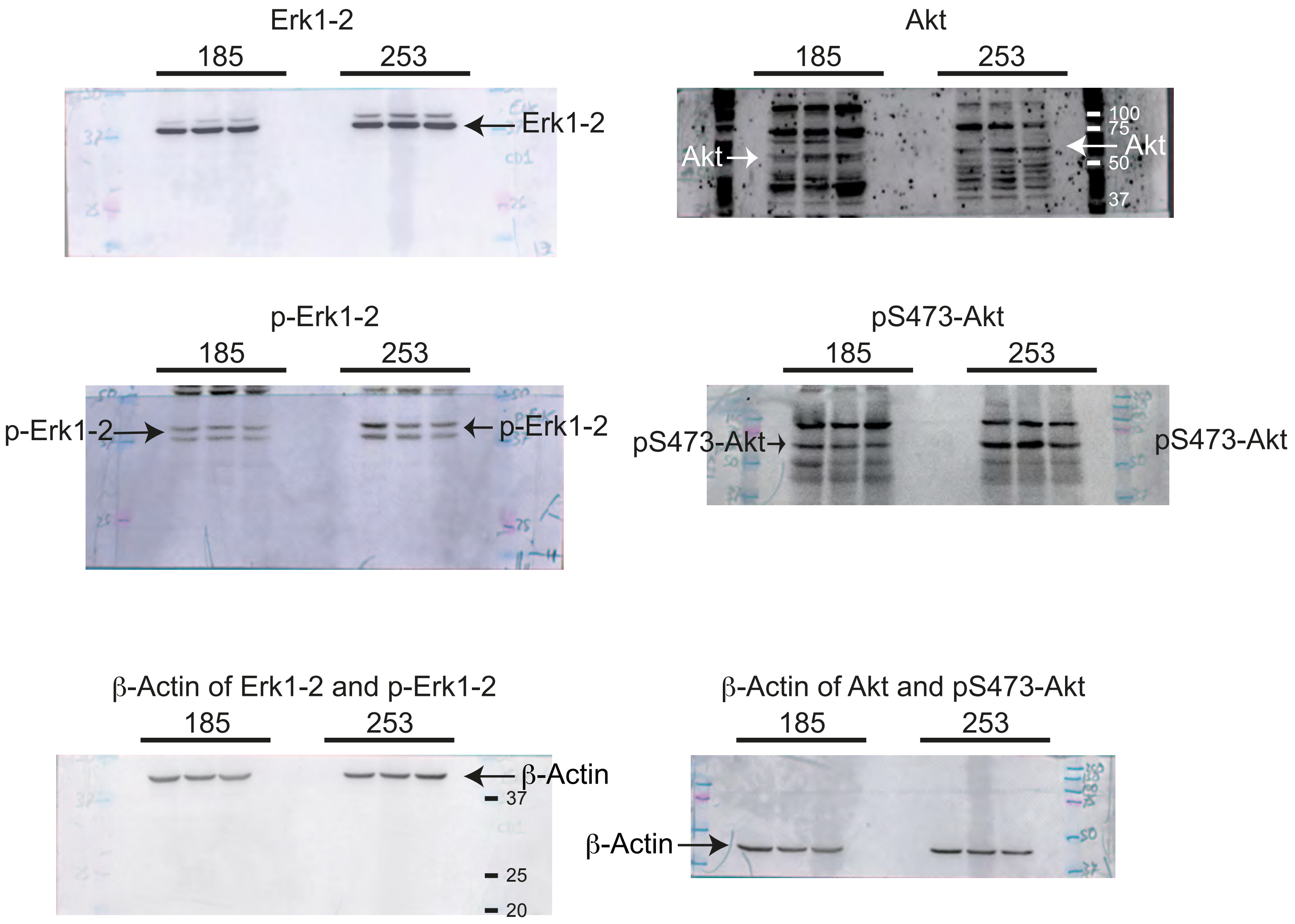

Figure S4 – Uncropped immunoblot images - continued

Unprocessed images for Supplementary Figure S3

Fak

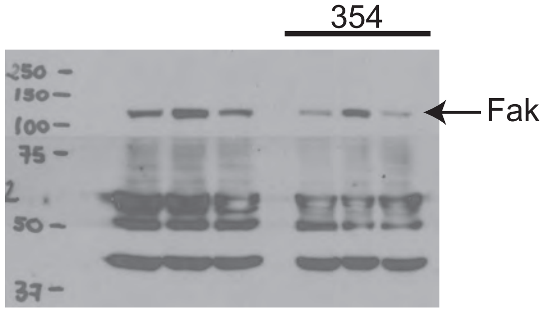

pY418-Src and pY925-Fak

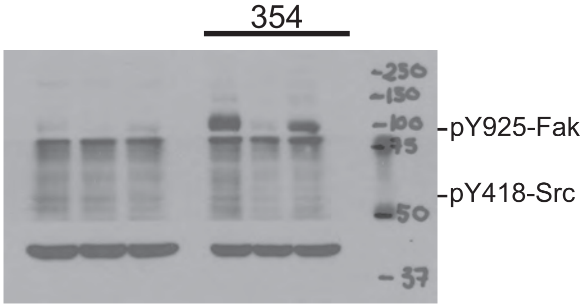

Src2

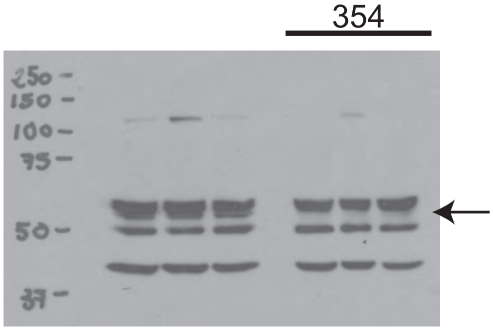

β-Actin of pY418-Src and pY925-Fak

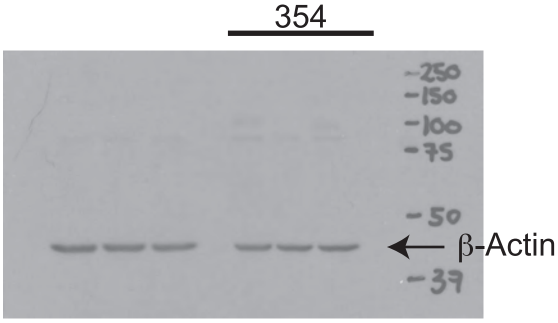

β-Actin of Src2 and Fak

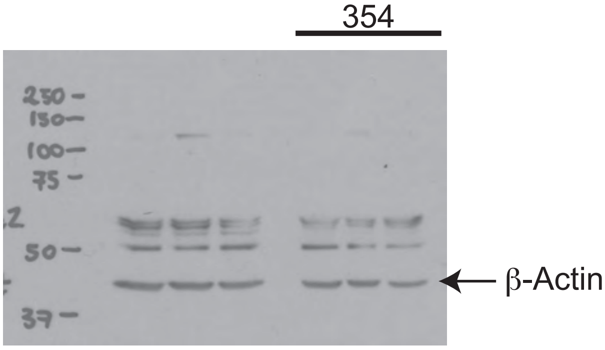

Supplement: Supplementary file 1 [file ijms-21-07437-s001.pdf]
